# Supplementary material for: Multimodal MALDI imaging mass spectrometry for improved diagnosis of melanoma
Source: PLoS One. 2024 May 31;19(5):e0304709. doi: 10.1371/journal.pone.0304709 (PMC11142536; doi:10.1371/journal.pone.0304709)
Supplement: S1 File — Results of the nested cross-validation on the training dataset and the fine tuned hypermeters. (PDF) [file pone.0304709.s001.pdf]

Supplementary file 1

In this work, we used the Python package *sklearn.linear_model.SGDClassifier* to implement the linear SVM model. Parameter *loss*=`hinge' (gives linear SVM), *alpha* is the regularization constant, *l1_ratio* is the Elastic Net mixing parameter, with 0 <= *l1_ratio* <=1. When *l1_ratio* = 0 corresponds to L2 penalty (standard regularizer for linear SVM models), *l1_ratio* = 1 to L1.

During the parameter tuning for the SVMs model, we used a grid search strategy, where the model exhaustively generates candidates from **a grid of parameter values** that are predefined by the user.

To efficiently keep track of the intermediate results during the nested cross-validation, we applied the open source platform MLflow[1] to manage the machine learning life-cycle. Specifically, we applied *mlflow.sklearn.autolog* for logging and loading of the scikit-learn models. Results of the nested cross-validation on the training dataset, and the fine tuned hyperparameters are shown below:

- Nested cross-validation results of unimodal IMS pipeline:

Outer fold	best alpha	best l1 ratio	best inner cv score-AUC	outer cv test mean AUC	outer cv test mean F1	outer cv test mean Precision	outer cv test mean Recall
1	1.00E-05	0.5	0.895	0.940	0.902	0.959	0.852
2	1.00E-09	1	0.913	0.807	0.679	0.853	0.563
3	1.00E-05	0.5	0.897	0.953	0.844	0.874	0.815
4	1.00E-05	0.5	0.905	0.911	0.832	0.826	0.837
5	1.00E-09	1	0.910	0.956	0.893	0.922	0.866
6	1.00E-05	0	0.915	0.911	0.784	0.933	0.676
7	1.00E-09	0.5	0.910	0.963	0.856	0.794	0.929
8	1.00E-05	0	0.912	0.899	0.800	0.755	0.850
9	1.00E-09	0.5	0.908	0.864	0.773	0.682	0.890
10	1.00E-05	0	0.910	0.948	0.864	0.863	0.866
	**Mean**		0.907	0.915	0.823	0.846	0.815
	**St.d**		0.007	0.049	0.067	0.085	0.110

- Nested cross-validation results of unimodal microscopy pipeline:

Outer fold	best alpha	best l1 ratio	best inner cv score-AUC	outer cv test mean AUC	outer cv test mean F1	outer cv test mean Precision	outer cv test mean Recall
1	0.00001	0	0.932	0.962	0.910	0.968	0.859
2	0.00001	0	0.941	0.887	0.839	0.807	0.873
3	0.00001	0	0.938	0.902	0.758	0.778	0.738
4	0.00001	0.5	0.935	0.933	0.815	0.882	0.758
5	0.00001	0	0.931	0.959	0.766	0.943	0.645
6	0.00001	0	0.941	0.915	0.797	0.919	0.703
7	0.00001	0	0.927	0.977	0.757	0.609	1.000
8	0.000001	0	0.933	0.925	0.833	0.897	0.777
9	0.00001	0.5	0.936	0.951	0.812	0.883	0.752
10	0.00001	0	0.928	0.963	0.913	0.882	0.945
	**Mean**		0.934	0.937	0.820	0.857	0.805
	**St.d**		0.005	0.030	0.056	0.104	0.111

1 https://github.com/mlflow/mlflow

- Nested cross-validation results of multimodal IMS and microscopy pipeline:

Outer fold	best alpha	best l1 ratio	best inner cv score-AUC	outer cv test mean AUC	outer cv test mean F1	outer cv test mean Precision	outer cv test mean Recall
1	1.00E-06	0	0.954	0.980	0.731	1.000	0.576
2	1.00E-05	0	0.967	0.919	0.829	0.886	0.780
3	1.00E-05	0	0.962	0.964	0.850	0.875	0.826
4	1.00E-05	0	0.960	0.977	0.887	0.974	0.815
5	1.00E-05	0	0.960	0.987	0.922	0.954	0.892
6	1.00E-05	0	0.971	0.939	0.850	0.945	0.772
7	1.00E-09	1	0.958	0.996	0.919	0.850	1.000
8	1.00E-09	1	0.962	0.968	0.888	0.863	0.915
9	1.00E-05	0	0.953	0.975	0.888	0.906	0.871
10	1.00E-09	0.5	0.962	0.976	0.898	0.952	0.850
	**Mean**		0.961	0.968	0.866	0.920	0.830
	**St.d**		0.005	0.023	0.056	0.051	0.112
